# Shadow Stadia and the Circular Economy

**DOI:** 10.3389/fspor.2022.937243

**Published:** 2022-07-05

**Authors:** Taryn Barry, Daniel S. Mason, Lisi Heise

**Affiliations:** ^1^Faculty of Kinesiology, Sport, and Recreation, Edmonton, AB, Canada; ^2^City of Edmonton, Urban Planning, Edmonton, AB, Canada

**Keywords:** sport facilities, stadiums, arenas, shadow stadia, circular economy

## Abstract

Most attention on stadium or arena-anchored development projects is placed on the scope and construction of the new sports facility, while less emphasis is on the facility left behind, which we describe as *shadow stadia*. Some *shadow stadia* are repurposed for mixed use development, others are demolished but have delayed redevelopment plans, while some remain abandoned and empty for years after the professional sports team or event is no longer present in the facility. The environmental impacts of *shadow stadia* are not fully understood, as limited research exists on how the immediate neighborhood anchored by pre-existing venues cope in the shadows of these new development plans and the loss of a sport venue and its events. Green strategies such as the circular economy may extend the lifecycle of existing sport faciltiies. To contribute to this discussion further, this perspective article will first discuss current advances in the academic literature on the circular economy. Second, it will present a comprehensive categorization of shadow stadia globally and future opportunities on integrating circularity into best practices. By doing so, this perspective article highlights several areas of future investigation that should be considered and planned for when major league sports teams and city leaders move their team and build new facilities.

## Introduction

Stadium or arena-anchored development projects remain an important part of urban redevelopment planning worldwide (Johnson and Whitehead, 2000; Crompton, 2004; Rosentraub, 2006). It has been argued that urban redevelopment through sport stadium or arena projects results in both positive and negative outcomes for cities and their residents (Rosentraub, 2009, 2014; Grant Long, 2013). However, while most attention is placed on the planning, scope, and construction of the new sports facility, less emphasis is on the facility or space left behind. *Shadow stadia* are vacated sites or venues such as arenas and stadiums once occupied by major professional or amateur sports franchises. When a facility is deemed obsolete by a team and its owners, it is effectively at the end of its lifecycle. When claiming obsolescence, team owners may decry the building unsafe or unfit (Unger, 1985; Holstege, 2017), that their revenue expectations are not being met (Shapiro et al., 2012), or the facility is lacking modern upgrades to maintain it as a state-of-the-art facility (deMause, 2016).

Shadow stadia offer both challenges and opportunities for local governments, developers, and communities once sports venues are vacated. Options to renovate or to build a new facility are often debated amongst owners of sport franchises. Some sites are only temporarily shadow stadia since they are repurposed for different development, while others are demolished or remain abandoned for years after facility use ceases.

Although the merits of urban redevelopment through sport stadia have been the subject of study and debate, a focus is typically placed on the impacts of a new stadium or arena development on its associated community. Thus, there is scant empirical research examining the impact of shadow stadia or the site to their respective communities. In addition, the outgoing site is not typically incorporated into the broader analysis meant to provide a comprehensive outlook of the economic, social, or environmental impacts to local communities—resulting in a lack of insight and empirical data to draw from.

This is an important issue as, more broadly, building and construction counts for 39% of carbon emissions worldwide (UN Environment and International Energy Agency, 2017), while operational emissions from energy used to cool, heat, and light buildings accounts for 28% of global carbon emissions (World Green Building Council, 2022). With this awareness, there is an emerging trend in stadium or arena design and construction to capitalize on environmental sustainability initiatives and gain either the International Organization for Standardization (ISO) 14001 Environmental Management certification or Leadership in Energy and Environmental Design (LEED) certification. Academic research focused on new stadia and urban development has also expanded recently (Mallen and Chard, 2012; Kellison et al., 2015; Triantafyllidis et al., 2018). For instance, Kellison and Hong (2015) identified growing pressure faced by architects and sport franchise owners to incorporate environmentally sustainable features into new stadia design, and found that economic savings over the lifespan of the facility is a key driver in the adoption of pro-environmental architecture and design. However, we are not aware of related published research on the potential environmental impacts of shadow stadia.

Growing pressure to incorporate green construction practices is a result of the increasing acceptance that the sport industry has a responsibility to environmental sustainability and climate justice. Sport's contribution is even recognized in the United Nations' 2030 Agenda for Sustainable Development, a plan of action for the planet, people, and prosperity (United Nations, 2022), and its Sustainable Development Goals (SDGs). The Agenda states (United Nations, 2022):

Sport is also an important enabler of sustainable development. We recognize the growing contribution of sport to the realization of development and peace in its promotion of tolerance and respect and the contributions it makes to the empowerment of women and of young people, individuals and communities as well as to health, education and social inclusion objectives (2030 Agenda for Sustainable Development A/RES/70/1, paragraph 37).

Two Sustainable Development Goals that sport can strive to meet include, SDG 11—make cities inclusive, safe, resilient and sustainable, and SDG 12—sustainable consumption and production (Schröder et al., 2019). There are many ways the sport industry can meet the objectives of the 2030 Agenda and these two SDGs. One innovative approach is moving toward emerging circular sustainability and construction methods. Wergeland and Hognestad (2021) recently emphasized green strategies and perspectives such as the circular economy that could extend the lifecycle of existing football stadia. Bengtsson et al. (2018) argued that the circular economy, defined through specific actions and practices such as eco-design, reuse, refurbishment, remanufacturing (Nasr and Thurston, 2006), repair and product sharing (Chertow and Ehrenfeld, 2012), can contribute to reaching the social, economic, and environmental targets set forth by the 2030 Agenda's Sustainable Development Goals.

As such, the following adopts a circular economy approach to examine and plan for future stadium or arena construction and their shadow stadia. It provides a brief overview of redevelopment trends that have emerged in cities challenged to solve blights created by vacant shadow stadia. By doing so, this perspective article highlights several areas of future investigation that should be considered and planned for when major league sports teams and city leaders move their team and build new facilities.

## Shadow Stadia and the Circular Economy

The circular economy (CE) approach has been gaining recognition and consideration from multinational companies (Lacy et al., 2014). There is also an increase in published researched on the CE in both the natural and social sciences (Kirchherr and van Santen, 2019). Increased research on the subject over the last decade was arguably ignited by the Ellen MacArthur Foundation report in 2012 (Geissdoerfer et al., 2017) that called for a new economic model to address the proliferation of world-wide resource depletion (Ellen MacArthur Foundation, 2012).

Broadly speaking, CE is an economic system whereby all types of waste are reduced through the continuous use of resources (Lacy et al., 2020). According to the Ellen MacArthur Foundation (2021), CE replaces the traditional Linear Economy that wastes materials once they have been used. CE is also based on the preservation and enhancement of natural capital, planning out waste, and lengthening the circulation of materials and products. CE is thereby defined through specific actions and practices such as eco-design, reuse, refurbishment, remanufacturing (Nasr and Thurston, 2006), repair and product sharing (Chertow and Ehrenfeld, 2012; Schröder et al., 2019).

From an urban development perspective, Corona et al. (2019) defined the concept of circular economy as a systematic approach to address and decrease urban sustainability issues through optimization of materials and energy. Kirchherr et al. (2017) explained CE as keeping energy and material flows within consumption and production by using long-lasting design, material and energy efficiency, by reusing and remanufacturing rather than recycling. Reusing can be defined as using a product several times without changing the design or material, while remanufacturing explains how a used product is renovated or maintained to the same standard as the new or original product. Recycling is where a product is destroyed to be used for manufacturing new products (Glader, 2019).

The urban construction sector is among the most evident sectors that contribute to negative environmental impacts (Kucukvar and Tatari, 2012, 2013; Onat et al., 2014) and research has shown that CE concepts such as the adaptive repurposing of existing, abandoned, and historical buildings, is beneficial for the environment and neighborhood revitalization (Bullen and Love, 2010; Baker et al., 2017; Foster, 2020). Drawing from concepts such as adaptive reuse and maintenance architecture, Wergeland and Hognestad (2021) concluded football (soccer) stadia have potential for circularity if the broader sports community becomes more willing to preserve architectural legacy. To do so, they argued further investment in stadium restoration is required to limit demolition of historic stadia, while legislative changes are necessary to make it easier to modify existing sports venues.

There are few studies that analyze circular approaches to sport stadia. Al-Hamrani et al. (2021) contributed to the first economic impact study of a circular application in the construction of Eduction City, a stadium built for the 2022 FIFA World Cup in Qatar. Rather than using a conventional concrete casting approach for the foundation of the new stadium, a cyclopean concreate methodology was employed, using a low-cost alternative material from existing waste products. The results of an environmental life cycle assessment found a 32% reduction in greenhouse gases. Meanwhile, Kucukvar et al. (2021) conducted a comprehensive analysis on Ras Abu Aboud (RAA), a reusable stadium for the 2022 FIFA World Cup, made out of modular shipping containers that can be dismantled and relocated after the event. The authors discussed how the social sustainability aspects of the circular economy can have a post-event legacy, suggesting that a circular design can save up to 60% of human health impacts and decrease dependency on imported construction materials. Nonetheless, more empirical research is necessary so local governments, the development industry, policy makers, community advocates, and academics understand the environmental implications of shadow stadia and the benefits of the CE. This research could better inform the process and assessment of new stadium development so as to meet SDGs 11 and 12.

## Redevelopment Trends in Shadow Stadia

Shadow stadia are the facilities that remain when sports franchises abandon them for new ones. For this study, information on stadia and arenas home to professional hockey, basketball, football, soccer, rugby, or cricket teams over the last 110 years around the world was collected and analyzed to address how cities address their shadow stadia. Olympic venues, motor-specific tracks, war purpose venues, or renovated structures were excluded from the analysis. Primary sources included press releases, council reports, first-hand accounts, legal documents, sports league Bylaws and Constitutions, magazines, newsletters, blogs, and data collected from Google Maps regarding the shadow stadia's recent physical state. The analysis also included academic studies, articles from major newspapers, and periodicals for secondary information on development trends. Through the analysis, 283 shadow stadia across 22 countries were found and collated. The redevelopment plans for shadow stadia were categorized in seven ways, including: *mixed-use redevelopment, grocery/retail, residential, replacement stadia, and infrastructure on the existing site, community facilities, site vacancies*, and *other*. Each category is described in more detail while including future circularity imaginaries for shadow stadia site redevelopment planning. Table 1 illustrates this categorization in detail.

**Table 1:** Redevelopment trends in shadow stadia.

**Country**	**Categories**						
	**Mixed-use**	**Grocery/retail**	**Residential**	**Use of existing land**	**Non-profit organizations**	**Vacant sites**	**Other**
Argentina		1					
Australia	1		3				
Austria	1		1				
Belgium						1	
Canada	2	1		1	1		1
England	9	15	44	1	5	7	7
France						1	
Germany	2		4			2	
Ireland			3		1	1	
Italy						1	
Japan		3			2		
Netherlands	2		5		1		
New Zealand	1		1			2	
Northern Ireland		1					
People's Republic of China		2					
Scotland		4	3		1	2	
Slovakia	1					1	
South Korea	1						
Spain	1	1	4		1		2
Switzerland						3	
United States of America	14	14	9	18	36	10	17
Wales	1		2				
Total	36	42	79	20	48	31	27

### Mixed-Used

Mixed-use redevelopment plans comprised of a combination of residential, retail, entertainment, and community recreation/green space have become increasingly favorable options for cities. Of the shadow stadia examined worldwide, 36 of those sites' repurposing plans included some application of mixed-use development strategy. While the mixed-use redevelopment has occurred with less frequency than other categories noted in these results, twelve countries are represented. One way these sites can integrate CE features is to use the materials from the demolition of the old facility, for example the bricks and other materials from the original site could be used in the new design. Not only can this support circularity, but an added benefit is it can contribute to the urban landscape and retain the city aesthetic.

### Grocery/Retail

Forty-two stadia were found to have involved a partnership with or received funding from a major grocery/retail chain as a part of the redevelopment of the site. This funding supported the relocation of the team, the construction of the new site, or both. Most redevelopment plans anchored by grocery chains have been concentrated primarily in the United Kingdom. One way to incorporate elements of circularity into a grocery/retail redevelopment site is to repurpose the original building or arena, rather than demolishing it. However, this requires the original building to be intact so that it can be efficiently refurbished. For future sport stadia construction projects, new facilities should be designed and built with the consideration of refurbishment, so that decades later a circular approach can be employed.

### Residential Development

A larger proportion of sites surveyed are categorized as residential developments, where seventy-nine sites which were repurposed for housing, whether it be apartment-style condominium towers, row housing, or community housing for low-income residents or senior citizens. Residential site development can also be emboldened to use CE elements in their creative planning, like mixed used development sites previously discussed. For example, the cement from the original sports facility can be remanufactured and incorporated into the foundation of the residential development site.

### Use of Existing Land

Many municipalities and franchise owners choose to rebuild an expanded modernized facility on the pre-existing site, or on an adjacent piece of land. Twenty stadia were either being built on an adjacent piece of land while the existing site was then converted into parking or other infrastructure facilities, or the existing stadium was demolished and the new stadium rebuilt on the site while its team found a temporary home during construction. One reason for this is often attributed to a lack of space large or central enough to accommodate a new stadium in an already dense urban area. Reusing the existing land may have circularity elements already associated with this redevelopment trend, although this does not determine whether there are other negative environmental impacts associated with the reuse of land, such as increased traffic congestion. This may occur due to lack of pubic consultation on changes to natural green spaces and existing/adjacent site redevelopment planning.

### Non-profit Organizations

Non-profit and charitable organizations have also benefitted from shadow stadia redevelopment opportunities. Forty-eight sites reviewed are categorized as having been transformed into public parks or community recreation areas, high school or post-secondary facilities, or donated to non-profit or charitable organizations. CE can be incorporated in this trend. For example, with reconfiguration, some outdoor stadia once used for professional football and baseball can be reused to hold local youth sport competitions.

### Vacant Sites

Thirty-one redevelopment projects experienced periods of vacancy that resulted in underutilized spaces, often in dense urban areas. These represent overlooked opportunities for tax revenue generation, and often require the support of social and emergency responder services to deal with human and infrastructure-related encounters. Often, vacant shadow stadia are a temporary situation; however, many stadia have experienced longer durations of uncertainty while negotiations took place between municipal government, lawyers, and community groups. The temporality of vacant sites may not advance the CE approach. Whether it is boarded-up stadia or unoccupied land after original buildings are demolished; vacant shadow stadia illustrate poor planning that may have negative social, economic, and environmental impacts on the nearby community.

### Other

Not all sites compiled fit into categories summarized above. Twenty-seven sites were converted into the following: commercial buildings, corporate headquarters, industrial yards, government buildings, public or private hospitals, major roadways, military barracks, hospitals, parking lots, or storage facilities.

In classifying shadow stadia and providing additional clarity regarding the complex challenges posed by shadow stadia, this perspective article has identified a subsequent circularity action to be facilitated by urban redevelopment stakeholders both internal and external to the sport industry. This will be discussed in more detail below.

## Discussion

This article sought to classify redevelopment trends that have emerged from cities challenged to solve blights created by vacant shadow stadia. The classification discussed above is an important first step in understanding, defining, and explaining the types of shadow stadia that exist worldwide. In doing so, it provides context for discussing and providing potential ways forward for the complex environmental challenges posed by shadow stadia.

This paper highlights several areas that necessitate future investigation and could be the basis for future research and action. First, we reiterate the recommendation from Rosentraub (2009) that in stadium or arena anchored urban redevelopment, planning must be large in scale and purposeful, with a combination of both public and private funding and input, as opposed to an expectation that an urban area requiring revitalization will occur over time organically. Rosentraub's (2009) recommendation also extends to environmental sustainability—it must include strategic planning for long term successful outcomes.

Second, reusing materials from demolition in the redevelopment plans of shadow stadia is vital to reduce the environmental impact of construction and building materials being wasted. As such, the stadium or arena anchored urban redevelopment industry should adopt a circular economy approach when permitted. Decisions on planning for the repurposing of old arenas and stadia must be done prior to a professional sport franchise moving locations, and the city must be facilitating and driving this dialogue and planning. Furthermore, sport franchises, their owners, and master developers must be held financially accountable for any failed shadow stadia redevelopment plans and potential environmental risks.

Third, we propose a modified circular economy approach be considered, whereby race, class, culture, and gender intersect with the environmental impacts of where people live, work and play. To do so, an environmental justice lens may be useful as a problem-framing tool to diagnose what the problems are and who is responsible for them, so a process of change can be established (Walker, 2012). One way this can be accomplished is by way of participation, where community members have access to the decision-making processes of local politicians and urban planners, through open dialogue and the opportunity for consultation (Schlosberg, 2007).

Fourth, an intersectional environmental lens that incorporates non-Western and Indigenous circularity discourses about sustaining or restoring natural life cycles (Friant et al., 2020) may be appropriate in differing global and local contexts. As such, lived experiences of residents may be gathered to examine potential concerns such as rising real estate prices, taxes, and gentrification in the surrounding neighborhood, since the “circularity discourse is not universal” (Wuyts and Marin, 2022, p. 11).

Fifth, we highlight the other potential environmental impacts that are created or maintained by using a CE approach. For example, the location of a shadow site could lead to a reduction in carbon emissions with new development and use of the site. For example, many of these shadow stadia are located on public transportation grids already, so when the site is being redeveloped the existing transit system could be incorporated into the new design, and ultimately lead to shorter commutes and decreased pollution, especially where new housing and density is created.

In conclusion, this article aimed to fill a gap in the literature by defining and examining shadow stadia; in doing we argue that circularity should be seriously considered and incorporated into planning when shadow stadia are left behind. To better understand the environmental and quality of life impacts of the communities living nearby shadow stadia, we recommend the approach of the circular economy should be explored in future research using the exemplars mentioned above.

## Data Availability Statement

The original contributions presented in the study are included in the article/supplementary material, further inquiries can be directed to the corresponding author/s.

## Author Contributions

LH collected and analyzed the data. TB and DM wrote the article. All authors contributed to the article and approved the submitted version.

## Conflict of Interest

The authors declare that the research was conducted in the absence of any commercial or financial relationships that could be construed as a potential conflict of interest.

## Publisher's Note

All claims expressed in this article are solely those of the authors and do not necessarily represent those of their affiliated organizations, or those of the publisher, the editors and the reviewers. Any product that may be evaluated in this article, or claim that may be made by its manufacturer, is not guaranteed or endorsed by the publisher.
